# Proximal Sensing of Soil Electrical Conductivity Provides a Link to Soil-Plant Water Relationships and Supports the Identification of Plant Water Status Zones in Vineyards

**DOI:** 10.3389/fpls.2020.00244

**Published:** 2020-03-11

**Authors:** Runze Yu, S. Kaan Kurtural

**Affiliations:** Department of Viticulture and Enology, University of California, Davis, Davis, CA, United States

**Keywords:** plant water status, soil electrical conductivity, spatial variability, selective harvest, anthocyanins, precision viticulture

## Abstract

The majority of the wine grapes are grown in Mediterranean climates, where water is the determining factor for grapevine physiology and berry chemistry. At the vineyard scale, plant water status is variable due to the variability in many environmental factors. In this study, we investigated the ecophysiological variability of an irrigated Cabernet Sauvignon (*Vitis vinifera* L.) vineyard. We used equidistant grid sampling to assess the spatial variations of the plants and soil, including plant water status by stem water potential (Ψ_*stem*_), leaf gas exchange, and on-site soil analysis. We also measured soil electrical conductivity (EC) by proximal sensing at two depths [0.75 – 1.5 m (sub soil); 0 – 0.75 m (top soil)]. Ψ_*stem*_ integrals were calculated to represent the season-long plant water status. On the base of realized Ψ_*stem*_ integrals, the vineyard was delineated into two functional homogeneous zones (fHZs) with one severely water stressed zone and one moderately water stressed zone. Sub soil EC was directly related to Ψ_*stem*_ (*r*^2^ = 0.56) and *g*_*s*_ (*r*^2^ = 0.39) when the soil was proximally sensed at harvest in 2018. Although the same trend was evident in 2019 we could not deduce a direct relationship. The fruits from the two fHZs were harvested differentially. Comparing the two fHZs, there was no significant difference in juice total soluble solids or pH. The severely water stressed zone showed significantly higher malvidin and total anthocyanins on a dry skin weight basis, but lower peonidin, malvidin on a per berry basis in 2018. In 2019, there were more quercetin and total flavonols per berry in the severely water stressed zone. Overall, this study provided fundamental knowledge of the viability of managing spatial variability by delineating vineyard into distinct zones based on plant water status, and the potentiality of proximally sensed soil EC in the spatial assessment of plant water status and the supporting of vineyard management.

## Introduction

Plant water status is one of the major drivers affecting grapevine physiology (Smart and Coombe, 1983), and is a determinant of grape berry chemistry (Martínez-Lüscher et al., 2014a). When soil water availability cannot fully meet the needs of plant growth and development, it becomes an abiotic stressor of the plants. Many physiological processes are affected when plants undergo water stress. The overall plant growth will be induced into reproductive maturity and dormancy as opposed to vegetative growth by the upregulation in abscisic acid synthesis (Chaves et al., 2010; Tombesi et al., 2015; Bonfante et al., 2017). For grapevines specifically, water stress was shown to influence canopy development, canopy microclimate, yield, and berry composition (Santesteban et al., 2011; Escalona et al., 2015). It would decrease leaf stomatal conductance and net carbon assimilation, leading to a decline in photosynthetic output. By various agronomic practices, water stress can be controlled within a mild to moderate range in red skinned wine grape cultivars. This may have beneficial effects on berry chemistry because water stress would suppress the grapevine vegetative growth being as a competing process for limiting photosynthetic resources (Intrigliolo and Castel, 2010). The assimilated carbohydrates are then repartitioned into berries and thus increasing total soluble solids (TSS) in the berries under moderate water stress, favoring the reproductive growth of the grapevines.

Vineyard systems are not uniform due to the existing spatial variability in growing site topography and soil characteristics (Brillante et al., 2017). Furthermore, cultural practices are usually applied uniformly without taking into account the spatial variability in vineyards. Besides, the complexity in vineyard systems makes it challenging to individualize each of the existing spatial variability for making management decisions. More often than not this leads to variability in grape composition at harvest, where the composition of the final wine would be compromised (Bramley, 2005). Thus, there is a need for a more comprehensive and precise approach to assess, monitor, and manage these variabilities by treating the vineyard as a whole system.

Proximal sensing in precision viticulture may be used to assess and monitor the spatial and temporal variability to fulfill this need (Matese et al., 2015). In a vineyard, under the same climate condition, the processes involved in the soil-plant continuum and the atmosphere system are strictly influenced by the soil spatial variability. These would unavoidably lead to a spatial variability in plant water status and berry composition (Brillante et al., 2014, 2018; Tardaguila et al., 2018). Assessing soil has been investigated in previous precision viticulture studies (Gómez-Míguez et al., 2007; Costantini et al., 2010; Bonfante et al., 2011; Brillante et al., 2017). Electrical conductivity (EC) (or its reciprocal electrical resistivity) was used to assess many soil variables as it acts as a function of soil physical and chemical properties, such as soil texture, moisture content, solute concentration, and temperature (Bushman and Mehalick, 1989; Bittelli, 2011). Proximal soil sensing is rapid and non-invasive, which can be utilized as a tool for soil assessments. Many studies have implemented electromagnetic induction (EMI) sensing in their data acquisition methods besides some other approaches such as ground-penetrating radar (GPR), and time domain reflectometry (TDR). This approach was shown the capability to capture the integrated effect of soil moisture, soil salinity and soil texture, and their spatial and temporal variability with a relatively high temporal resolution and promptness in data acquisition (Hardie and Doyle, 2012; Brillante et al., 2014; Su et al., 2014). The ability to assess soil properties and plant water status more rapidly by proximal sensing is beneficial in commercial vineyards because it can provide the possibility to monitor and manage the spatial variability in soil responsively, that may further minimize the variations in final berry composition and wine chemistry (Brillante et al., 2018). Additionally, due to the significance of plant water status on berry chemistry, it is possible to evaluate the berry chemical composition once the relationship between soil electromagnetic properties and plant water status is determined.

For wine grape cultivars, flavonoid compounds constitute the most abundant class of berry secondary metabolites. They are critical in determining organoleptic properties in wine, such as color, flavor, mouth-feel, and also aging potential (Lorrain et al., 2013). The biosynthesis of these compounds are responsive to plant water status, where moderate water stress usually resulted in upregulation in flavonoid biosynthesis (Castellarin et al., 2007a). Managed water stress can contribute to a higher ratio of tri-hydroxylated over di-hydroxylated flavonoids due to the up-regulation of flavonoid 3′5′-hydroxylases (F3′5′H) (Castellarin et al., 2007b), which would enhance the compound stability against degradation (Liu et al., 2018). However, the higher concentration of flavonoids observed under water stress may be due to berry dehydration rather than alteration of flavonoid biosynthesis (Hardie and Doyle, 2012). Based on the captured variability in vineyards, vineyard delineation can be utilized to minimize the variability between zones pairing with targeted agronomic practices (González-Fernández et al., 2017). Selective harvest is one example of these practices when fruits are picked differentially, or segregated into various batches prior to the fermentation for producing wine with different rankings or characteristics (Bramley et al., 2011b; Priori et al., 2019). This approach can coalesce the variable ripening stages that may occur within the vineyard, where the relatively unripe fruits would be imparting unripe characteristics in the final wines (Parr et al., 2007). The variability in grape productivity and composition will always be present to a certain extent within vineyards. However, some prevailing faulty characters due to the uneven ripeness stages resulting from the heterogeneity in vineyards may impair quality, yielding undesired sensory properties in the final products (Kontoudakis et al., 2011). Hence, it is necessary to be able to minimize the variability within vineyards to achieve relatively the same maturity for vinification, and selective harvest can provide a direct way to satisfy this purpose.

Based on these previous studies, the objectives of this study were to investigate the variability observed in soil EC, and how it is translated into the variability in plant water status. Subsequently, this study investigated the relationship between proximal soil sensing and grape berry chemistry to bridge the gap between available sensing technologies and advanced chemical analysis methods. We also investigated whether the selective harvest approach, by delineating vineyard into different management zones based on plant water status, would minimize the variability in grape berry chemistry; and whether this zoning can be directed by proximal soil sensing, specifically by soil EC.

## Materials and Methods

### Vineyard Site, Plant Materials, and Weather

This study was conducted in a commercial vineyard with Cabernet Sauvignon grafted on 3309C (*V. riparia* × *V. rupestris*) in 2018 and 2019. This vineyard was located in Oakville, Napa County, California, United States. Grapevines were planted at 1.5 m × 2.0 m (vine × row), and trained as a bi-lateral cordon on a single high wire. The vineyard was pruned mechanically to a spur height of 100 mm with no further canopy management in 2018, and treated with mechanical shoot removal at E-L stage 17 to meet production demands. Irrigation was applied with a drip irrigation system with two 2L/h emitters at each plant, starting at fruit-set to harvest to replace 50% of crop evapotranspiration demand (ETc).

Weather data at the research site during the growing season was obtained from the California Irrigation Management Information System (CIMIS) station #77, in Oakville, CA which was 200 m away from the research site. Precipitation and reference evapotranspiration data were acquired to direct irrigation scheduling during the growing season. Applied irrigation amounts were calculated as the product of calculated crop coefficient and reference evapotranspiration. The crop coefficient was calculated as reported by Williams and Ayars (2005). Air temperature was acquired from the station for growing degree days (GDD) calculation.

### Experimental Design

**FIGURE 1:**
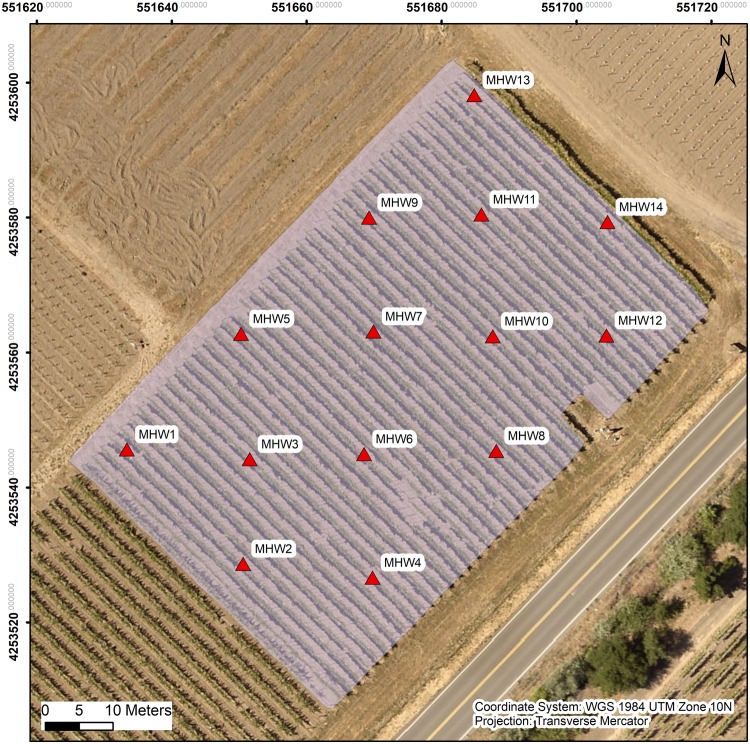
Map of the experimental block with 14 experimental units marked, red triangles illustrate the locations of the middle vine among the three vines in each experimental unit. “MHW” is the abbreviation for “mechanical high wire”, as named for the experimental block.

We used an equidistant 30 m × 30 m grid to sample and collect on-site measurements which contained 14 experimental units with 3 vines in each experimental unit. Geolocations of each center vine within each experimental unit were recorded with GPS unit (Yuma 2, Trimble Inc., Sunnyvale, CA, United States) connected to a Trimble Pro 6T DGNSS receiver (Trimble Inc., Sunnyvale, CA, United States) for further GIS analysis (Figure 1).

### Soil Property Assessment

Soil samples were taken on at field capacity from the two depths in the 14 experimental units, corresponding to the two depths that proximal soil sensing was conducted. Soil total organic matter (OM) and soil texture were measured according to the soil analysis methods in the North American Proficiency Testing (NAPT) program, Western states section. OM was measured by loss on ignition method (S – 9.10), soil texture was acquired by hydrometer analysis (S – 14.10). Soil gravel content was determined by section 26 in USDA Handbook No. 60 (Diagnosis and Improvement of Saline and Alkali Soils).

The instrument used for assessing bulk soil electrical conductivity was EM38-MKII (Geonics Ltd., Mississauga, ON, Canada) used in both vertical dipole mode and horizontal dipole mode to assess two depths [0.75 – 1.50 m (sub soil EC) and 0 – 0.75 m (top soil EC)] of measurements. The sensor of the instrument was calibrated according to the manufacturer’s instructions to minimize the errors before the survey. The instrument was placed on a PVC sled at an approximately 15 cm height above the ground, and pulled by an all-terrain vehicle along the inter-rows at a distance of about 0.5 m to avoid interference phenomena with the vehicle. The PVC sled made possible to keep the instrument at a constant distance from the soil surface, making data acquisition easier and more accurate. In both years, the soil EC measurements were assessed on the dates close to harvest, which occurred on 28 September 2018 and 20 September 2019.

### Plant Water Status, Gas Exchange and Yield Component Assessment

Mid-day stem water potential (Ψ_*stem*_) measurements were taken in 2018 and 2019 to assess plant water status. The measurements was assessed bi-weekly from 16 August 2018 and 29 May 2019. Three leaves in the shade were selected from the main shoot axis on the grapevines, and were concealed in pinch-sealed Mylar^§^ bags for about 2 h prior to the measurements in each experimental unit. A pressure chamber (Model 615D, PMS Instrument Company, Albany, OR, United States) was used to take the measurements. To summarize the temporal information assessed by Ψ_*stem*_ measurements, Ψ_*stem*_ integrals were calculated by using natural cubic splines (Myers, 1988). The sum of the values were divided by the number of the days between the first and the last measurements in each year to make the data comparable to each individual measurement.

In parallel with Ψ_*stem*_ measurements at mid-day, leaf gas exchange measurements were taken to assess leaf photosynthetic activities by using a portable infrared gas analyzer CIRAS-3 (PP Systems, Amesbury, MA, United States). The measurements were assessed bi-weekly from 16 August 2018 and 29 May 2019. Three sun-exposed leaves were selected from the main shoot axis in each experimental unit, and three readings were taken from each leaf. Gas exchange measurements were taken when the sunlight condition was close to saturating in both years (average *PARi* = 1713 ± 249 μmol m^–2^ s^–1^ in 2018, 1721 ± 206 μmol m^–2^ s^–1^ in 2019). The relative humidity was set at 40%, the reference CO_2_ concentration was set at 400 μmol CO_2_ mol^–1^ as the standard environmental condition setting in CIRAS-3. Net carbon assimilation rate (*A*_*N*_, μmol CO_2_ m^–2^ s^–1^) and stomatal conductance (*g*_*s*_, mmol H_2_O m^–2^ s^–1^) were obtained. Intrinsic water use efficiency (WUEi) was calculated as the proportion of *A*_*N*_ over *g*_*s*_ (μmol CO_2_ mmol^–1^ H_2_O). The *g*_*s*_ integrals were calculated to represent the long-term stomatal responses.

Leaf area index (LAI) was measured to characterize grapevine canopy growth, and converted into leaf area on 16 August 2018 and 15 August 2019 by a smartphone based program, VitiCanopy, coupled with an iOS system (Apple Inc., Cupertino, CA, United States) (De Bei et al., 2016). The gap fraction threshold was set to 0.75, the extinction coefficient was set to 0.7, and sub-divisions were 25. A ‘selfie-stick’ was used for easy access to place the device about 75 cm underneath the canopy. The device was positioned with the maximum length of the screen being perpendicular to the cordon, and the cordon being at the middle of the screen according to the user’s instruction (De Bei et al., 2016). In each experimental unit, three images were taken to capture half canopy of each vine, and analyzed by the software. Total leaf areas were calculated based on both LAI values and unit ground area in each experimental unit, and then the leaf area to fruit ratio was calculated.

All clusters in each experimental unit were harvested, counted, and weighed on a single harvest day in both seasons (27 September 2018 and 23 September 2019). Yield components were then calculated for assessing cluster number per vine, average cluster weight, berry number per vine, and yield per vine. Single berry weight was calculated by averaging total berry weights by total berry numbers from the collected berry samples.

### Berry Primary Metabolite Assessment

From each experimental unit 75 berries were randomly sampled, and were separated into two subsets with 55 berries and 20 berries individually. The set with 55 berries was used for berry primary metabolite analysis, including TSS, juice pH, titratable acidity (TA), and berry weight assessments. The set with 20 berries was for assessing dry berry skin weight and skin flavonoid contents.

Berry TSS was measured by a digital refractometer (Atago PR-32, Bellevue, WA, United States) and expressed as °Brix. Juice pH and TA were measured with an automated titrator (862 Compact TitroSampler, Metrohm, Switzerland) and expressed as g of tartaric acid per L of juice.

### Extraction of Skin Flavonoid Compounds

Skin tissues were manually removed from the subset of 20 berries with a scalpel, separated from the seeds and pulps, and lyophilized (Centrivap Benchtop Centrifugal Vacuum Concentrator 7810014 equipped with Centrivap −105°C Cold Trap 7385020, Labconco, Kansas City, MO, United States). Dry skin weights were recorded after lyophilization, and then the skin tissues were powderized with a mixing mill (MM400, Retsch, Mammelzen, Germany). We used 50 mg (±5% deviation allowed) of dry skin powder and mixed with 1 mL of methanol:water:7 M hydrochloric acid (70:29:1) to initiate the extraction at 4°C for 24 h. Then, the extracts were centrifuged at 5,000 rpm for 15 min, and the supernatants were separated from the sediments, filtered by PTFE membrane filters (diameter: 13 mm, pore size: 0.45 μm, VWR, Seattle, WA, United States), and transferred into HPLC vials before injection.

### Berry Skin Flavonoid Analysis

Skin anthocyanins and flavonols were analyzed by a reversed-phase HPLC (Agilent model 1260, Agilent Technologies, Santa Clara, CA, United States) consisting of a vacuum degasser, an autosampler, a quaternary pump, and a diode array detector with a column heater. A C18 reversed-phase HPLC column (LiChrosphere 100 RP-18, 4 × 520 mm^2^, 5 μm particle size, Agilent Technologies, Santa Clara, CA, United States) was used for the utilized method. The mobile phase flow rate was 0.5 mL min^–1^, and two mobile phases were used, which included solvent A = 5.5% aqueous formic acid; solvent B = 5.5% formic acid in acetonitrile. The HPLC flow gradient started with 91.5% A with 8.5% B, 87% A with 13% B at 25 min, 82% A with 18% B at 35 min, 62% A with 38% B at 70 min, 50% A with 50% B at 70.01 min, 30% A with 70% B at 75 min, 91.5% A with 8.5% B from 75.01 min to 90 min. The column temperature was maintained at 25°C. Detection of flavonols and anthocyanins was carried out by the diode array detector at 365 and 520 nm, respectively. A computer workstation with Agilent OpenLAB (Chemstation edition, version A.02.10) was used for chromatographic analysis.

All solvents used in this analysis were of HPLC grade, including acetonitrile, methanol, hydrochloric acid, formic acid purchased from Fisher Scientific (Santa Clara, CA, United States). Standards used for compound identification included malvidin 3-*O*-glucoside purchased from Extrasynthese (Genay, France), myricetin-3-*O*-glucuronide, myricetin 3-*O*-glucoside, quercetin 3-*O*-glucunoride, quercetin 3-*O*-galactoside, quercetin 3-*O*-glucoside, kaempferol 3-*O*-glucoside, isorhamnetin 3-*O*-glucoside, and syringetin 3-*O*-glucoside purchased from Sigma-Aldrich (St. Louis, MO, United States).

### Statistical Analysis

Geostatistical analysis and kriging for soil EC were performed by using package gstat 1.1-6 (Pebesma, 2004). Due to the nature of proximal sensing, there were many outliers captured when assessing soil EC. The data were filtered by Tukey’s rule to remove outliers of soil EC either below the first quartile by 1.5 inter-quartile range, or above the third quartile by 1.5 inter-quartile range. To further remove the outliers, the data were filtered by the speed that the vehicle was driving, which was between 3.2 km per hour to 8 km per hour. Variograms were assessed by automap package 1.0-14 (Hiemstra, 2013), and fitted to perform kriging. The specific soil EC values were extracted from the location of each experimental unit, these EC values were further used to performance correlation analysis.

Kriging was performed in ArcGIS (version 10.6, Esri, Redlands, CA, United States) and *k*-means clustering was performed in R (RStudio, Inc., Boston, MA, United States) with package NbClust, v3.0 (Charrad et al., 2014). An ordinary kriging method was used since there was no trend observed in the vineyard. In 2018, a spherical semivariogram model was chosen with a major range of 70.963 m, a nugget of 0, and a partial sill of 0.039 after cross-validation. The cross-validation of the model showed a root mean square error (RMSE) of 0.132 MPa, an average standard error of 0.124 MPa. In 2019, the vineyard was delineated into two clusters as well. A spherical semivariogram model was chosen with a major range of 35.48 m, a nugget of 0, and a partial sill of 0.012 after cross-validation. The cross-validation of the model showed a root mean square error (RMSE) of 0.100 MPa, an average standard error of 0.100 MPa. *k*-means clustering analysis and the practical manageability were considered when delineating the vineyard. The vineyard was delineated into two clusters by *k*-means clustering based on Ψ_*stem*_ integrals based on its significant role in connecting soil to plant physiology, including a severely water stressed zone and a moderately water stressed zone. The separation described 70.8% in 2018 and 67.8% in 2019 of the variability in the plant water status according to the result of between sum of squares/total sum of squares. Based on this delineation, data from the experimental units finally grouped together according to their locations within each cluster for the statistical analysis comparing grapevine physiological and berry chemistry measurements.

Data were tested for normality by using Shapiro–Wilk’s test, and subjected to mean separation by using one-way ANOVA with the package “stats” in Rstudio (R Foundation for Statistical Computing, Vienna, Austria) (R Core Team, 2019). Significant statistical differences were determined when *p*-values acquired from ANOVA were of 0.05 or less. Linear regression analysis was performed by SigmaPlot 13.0 (Systat Software Inc., San Jose, CA, United States). The coefficient of determination between variables was calculated in linear regression analysis, *p*-values were acquired to present the significances of the linear fittings.

## Results

### Weather and Soil EC at Experimental Site

**FIGURE 2:**
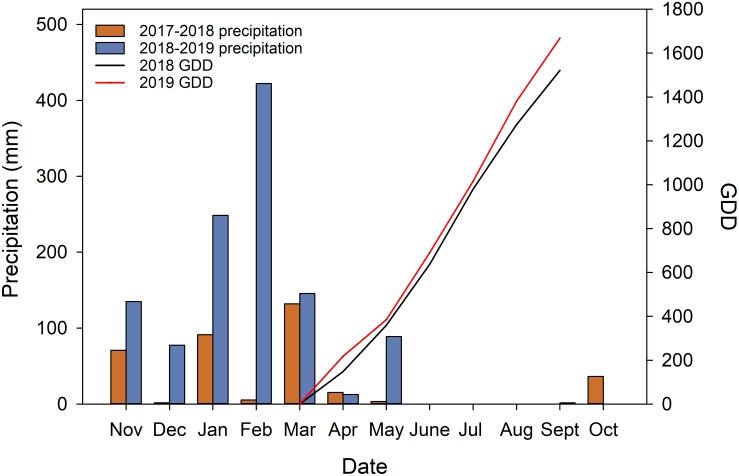
Weather data acquired from California Irrigation Management Information System (CIMIS) station (#77 Oakville, CA) and calculated growing degree days (GDD).

During the execution of the experiment, the precipitations received during the 2 years were vastly different (Figure 2). The experiment site received 356.2 mm and 1132.1 mm precipitation (from the previous November to October when fruits were harvested) in 2018, and 2019 respectively. Of the total precipitations received 88.71% of them were received during the dormant season in 2018 (from previous November to April). In 2019, 91.98% of the precipitation received was during the dormant season. The precipitation during the growing season was very limited. The research site only received 0.5 mm in 2018 and 1.7 mm in 2019 during the study time in each year from June to September. GDD accumulation showed that in 2019, the heat accumulation was higher in the second year with 1668.9°C compared to 1521.7°C in 2018.

**FIGURE 3:**
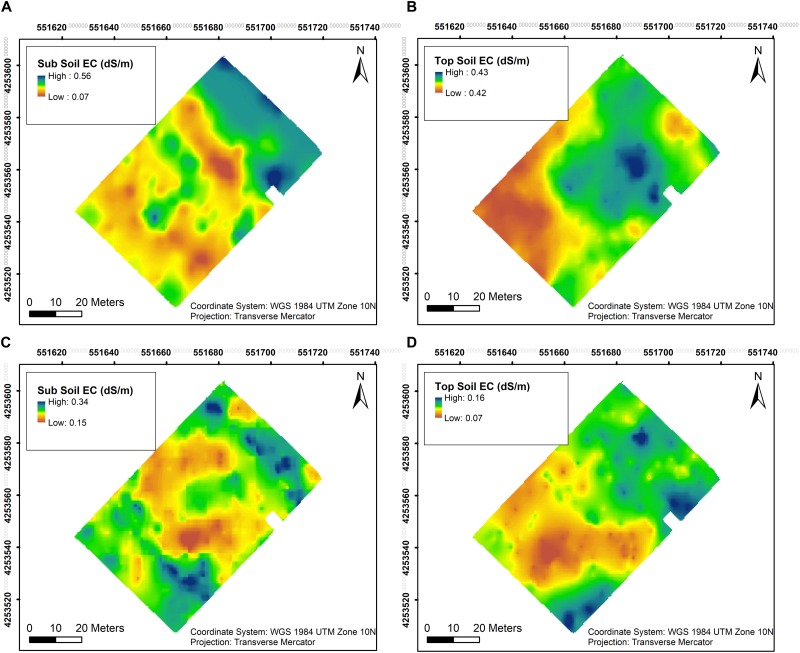
Interpolation maps of soil electrical conductivity (EC) in two depths assessed by EM38 in 2018 and 2019. **(A)** Sub soil EC in 2018, **(B)** top soil EC in 2018, **(C)** sub soil EC in 2019, and **(D)** top soil EC in 2019.

Soil EC was assessed at two different depths close to harvest in both years. In 2018, EC values in sub soil were generally lower with the lowest value at 0.07 dS/m in the southwestern section as well as the central section of the vineyard, the rest of the vineyard having higher EC with the maximum value of 0.56 dS/m (Figure 3A). Top soil EC values in the southwestern section of the vineyard in 2018 with 0.42 dS/m compared to 0.43 dSm in the rest of vineyard and did not vary appreciably in this year (Figure 3B). However, EC values in sub soil were lower only in the central section of the vineyard in 2019, showing the lowest value of 0.15 dS/m and highest of 0.34 dS/m in the rest of the vineyard (Figure 3C). A similar trend was evident in the top soil in 2019 compared to the first season, where lower EC values of 0.07 dS/m were observed in the southwestern section of the vineyard compared to 0.16 dS/m in the rest of the vineyard (Figure 3D).

### Plant Water Status, and Photosynthetic Activity

**FIGURE 4:**
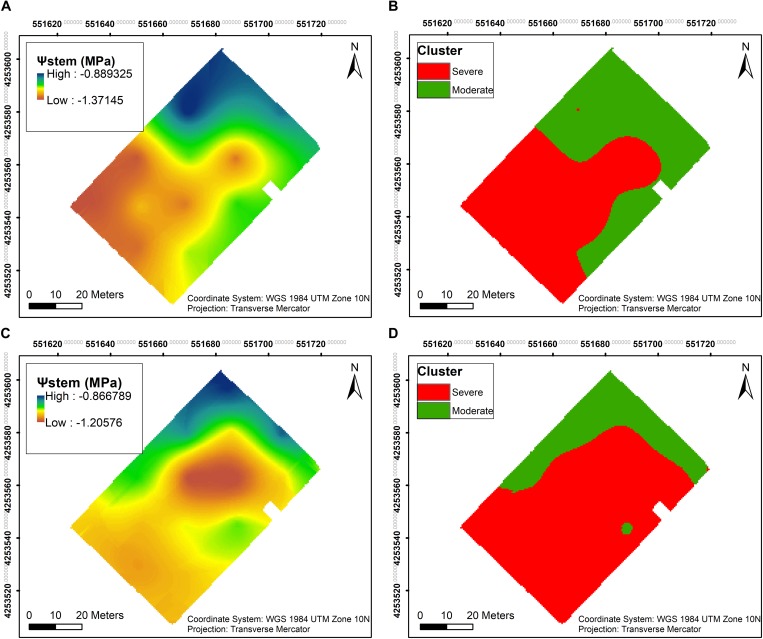
Interpolation maps of plant water status, presented as stem water potential (Ψ_*stem*_), and *k*- means clustering maps, delineating the vineyard into two functional homogeneous zones (fHZs) in 2018 and 2019. **(A)** Ψ_*stem*_ kriging map in 2018, **(B)** *k*- means clustering of Ψ_*stem*_ integrals in 2018, **(C)** Ψ_*stem*_ kriging map in 2019, **(D)** *k*- means clustering of Ψ_*stem*_ integrals in 2019.

Ψ_*stem*_ was measured throughout both seasons, and the overall trend in long-term Ψ_*stem*_ was able to partially elucidate the trend seen in the soil EC maps. Two clusters were calculated within the vineyard to delineate the whole block based on Ψ_*stem*_. A clear pattern was evident in 2018, where most of the southwestern section showed more negative Ψ_*stem*_ values than the rest of the vineyard (Figure 4A). In 2019, a larger area in the central section of the vineyard had more negative Ψ_*stem*_ values in the plants (Figure 4C). Comparing the clustering of both years, there was a 73.2% similarity between the two clusterings in 2018 and 2019 (Figures 4B,D).

**FIGURE 5:**
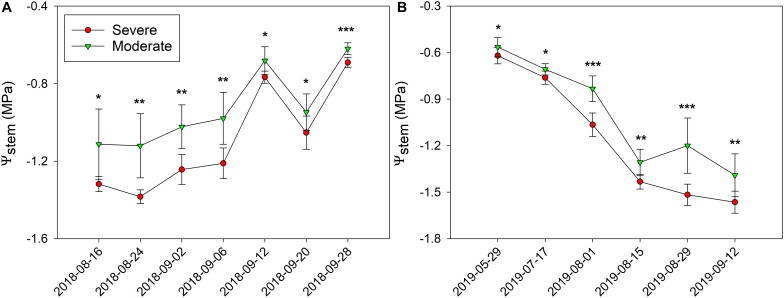
Progression of stem water potential (Ψ_*stem*_) between the two functional homogeneous zones (fHZs) in 2018 and 2019. Error bars represent standard deviation from the mean.

In 2018, Ψ_*stem*_ were consistently separated between the two fHZs (Figure 5A). The overall Ψ_*stem*_ values were consistent in 2018. However, there were exceptions to this trend on 20 September and 28 September due to the precipitation took place on 10 September and 23 September. The research site received 0.2 mm of precipitation during this period of time, causing Ψ_*stem*_ values to increase. In 2019, as the soil gradually dried, the Ψ_*stem*_ eventually became more negative throughout the season (Figure 5B). Between the two water status zones, Ψ_*stem*_ were also consistently separated in 2019, and a 0.27 MPa Ψ_*stem*_ difference was observed between these two fHZs in 2018, but a 0.18 MPa in 2019.

**FIGURE 6:**
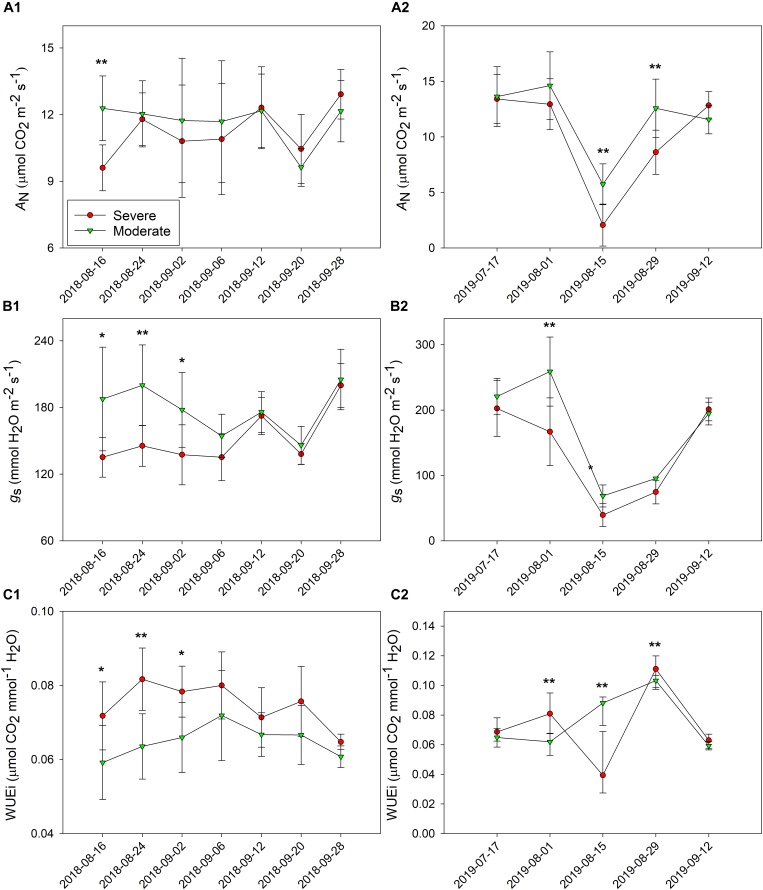
Progression of leaf gas exchanges between the two functional homogeneous zones (fHZs) in 2018 and 2019. **(A)** net carbon assimilation, *A*_*N*_, **(B)** stomatal conductance, *g*_*s*_, **(C)** intrinsic water use efficiency, WUE_i_, (1) 2018, (2) 2019. Error bars represent standard deviation from the mean.

*A*_*n*_ was measured in both years, and the separation between the two water status zones was not evident (Figures 6A1,A2). In 2018, the differences in Ψ_*stem*_ transiently translated into *A*_*n*_ between the two water status zones. We saw differences on 16 August 2018, 15 August 2019, and 29 August 2019, where the moderately water stressed zone had significantly greater *A*_*n*_. There was a drop in *A*_*n*_ and *g*_*s*_ values on 15 August 2019 due to an extreme weather condition the plants were experiencing with an ambient air temperature of 40.89 ± 0.50°C and a leaf temperature of 45.02 ± 1.48°C. However, this extreme condition did not affect the separations in gas exchange between the two fHZs except it showed an opposite result in WUEi.

The moderately water stressed zone had greater *g*_*s*_ when compared to the severely water stressed zone from 16 August to 2 September in 2018, but there was not difference on the other dates of that season (Figure 6B1). In 2019, the same differences between the two fHZs were observed only on 1 August and 15 August with moderately water stressed zone having higher *g*_*s*_ values (Figure 6B2).

In contrast to the *g*_*s*_, WUEi was greater in the severely water stressed zone within the same period of time in 2018 (Figure 6C1). In 2019, WUEi was significantly higher in the severely water stressed zone on 1 August and 29 August (Figure 6C2). On 15 August 2019, the moderately water stressed zone transiently had higher WUEi than the severely water stressed zone.

### Yield Components, Berry Composition, and Berry Skin Flavonoids

Yield components and berry primary metabolites were measured in both 2018 and 2019. In 2018, there was no difference observed in cluster number per vine, cluster weight, berry number per vine, or yield per vine between the two fHZs (Table 1). However, berry weight and berry skin weight were greater in the moderately water stressed zone compared to severely water stressed zone. There was no difference in leaf area, leaf area to fruit ratio between the two fHZs. The two fHZs had the same berry juice TSS, TA, and pH at harvest in 2018.

**TABLE 1:** Yield components and berry primary metabolites at harvest of Cabernet Sauvignon as separated by plant water status zoning in Oakville, CA in 2018 and 2019^a^.

		Cluster no. per vine	Cluster weight (g)	Yield per vine (kg)	Berry weight (g)	Skin weight (g)	Berry no. per vine	Leaf area (m^2^)	Leaf area/fruit (m^2^/kg)	TSS (°Brix)	TA (g L^–1^)	pH
2018	Severe Water Stress ± SD	110.22 ± 19.32	80.03 ± 16.69	8.45 ± 1.08	1.14 ± 0.07 **b**	0.05 ± 0.00 **b**	7387.6 ± 894.48	4.51 ± 1.09	0.55 ± 0.19	21.63 ± 1.22	9.43 ± 0.56	3.24 ± 0.03
	Moderate Water Stress ± SD	98.57 ± 26.78	90.71 ± 9.88	8.82 ± 2.15	1.29 ± 0.05 **a**	0.06 ± 0.01 **a**	6930.9 ± 1783.45	4.33 ± 0.59	0.51 ± 0.10	22.33 ± 1.66	9.45 ± 0.35	3.23 ± 0.04
	*p*-value	ns	ns	Ns	0.001	0.014	ns	ns	ns	ns	ns	ns
2019	Severe Water Stress ± SD	78.19 ± 17.02	61.35 ± 9.04	4.77 ± 1.18	0.98 ± 0.09	0.07 ± 0.01	5058.34 ± 1304.35	5.72 ± 0.93	1.26 ± 0.36	26.12 ± 1.28	8.29 ± 0.25 **b**	3.41 ± 0.13
	Moderate Water Stress ± SD	89.53 ± 21.95	67.01 ± 5.45	5.96 ± 1.31	1.03 ± 0.06	0.07 ± 0.01	5825.56 ± 1549.39	5.86 ± 0.62	1.01 ± 0.14	27.01 ± 1.23	8.92 ± 0.73 **a**	3.47 ± 0.15
	*p* value	ns	ns	ns	ns	ns	ns	ns	ns	ns	0.034	ns
Year		0.01571	<0.0001	<0.0001	<0.0001	<0.0001	<0.0001	ns	<0.0001	<0.0001	<0.0001	<0.0001
Year × Zoning		ns	ns	ns	ns	ns	ns	ns	ns	ns	ns	ns

*^a^ANOVA to compare data (p-value indicated); Letters within columns indicate significant mean separation according to Tukey’s HSD test.*

In 2019, there was no difference in any of the yield components either (Table 1). The fruits showed a more advanced maturity in 2019 compared to the first season. However, there was no difference observed in berry primary metabolites between the two water status zones except TA. The moderately water stressed zone had higher TA than the severely water stressed zone.

Skin flavonols were not generally affected by the spatial variations of plant water status. However, there were differences observed between the two water status zones in the total quercetin and total flavonols on a per berry basis, where the severely water stressed zone had higher quercetin and total flavonols in 2019 (Table 2). There was no difference observed in any other flavonol derivatives.

**TABLE 2:** Grape berry skin flavonols and anthocyanins at harvest of a Cabernet Sauvignon vineyard as separated by plant water status zoning in Oakville, CA in 2018 and 2019^a^.

		Myricetin		Quercetin		Kaempferol		Total flavonols		Delphinidin		Cyanidin		Petunidin		Peonidin		Malvidin		Total anthocyanins	
		mg g^–1^_skin dry wt_	mg berry^–1^	mg g^–1^_skin dry wt_	mg berry^–1^	mg g^–1^_skin dry wt_	mg berry^–1^	mg g^–1^_skin dry wt_	mg berry^–1^	mg g^–1^_skin dry wt_	mg berry^–1^	mg g^–1^_skin dry wt_	mg berry^–1^	mg g^–1^_skin dry wt_	mg berry^–1^	mg g^–1^_skin dry wt_	mg berry^–1^	mg g^–1^_skin dry wt_	mg berry^–1^	mg g^–1^_skin dry wt_	mg berry^–1^
2018	Severe Water Stress ± SD	0.87 ± 0.11	0.05 ± 0.00	1.05 ± 0.19	0.06 ± 0.01	0.28 ± 0.05	0.02 ± 0.00	2.78 ± 0.38	0.15 ± 0.01	8.62 ± 0.68	0.39 ± 0.05	1.06 ± 0.07	0.02 ± 0.00	6.13 ± 0.42	0.25 ± 0.03	2.89 ± 0.17	0.10 ± 0.01 **b**	40.07 ± 2.20 **a**	1.33 ± 0.11 **b**	58.77 ± 2.97 **a**	3.22 ± 0.24
	Moderate Water Stress ± SD	0.77 ± 0.13	0.05 ± 0.01	1.00 ± 0.33	0.06 ± 0.02	0.27 ± 0.05	0.02 ± 0.00	2.56 ± 0.52	0.16 ± 0.03	8.02 ± 1.61	0.43 ± 0.12	1.06 ± 0.19	0.03 ± 0.02	5.62 ± 0.83	0.27 ± 0.06	3.04 ± 0.45	0.13 ± 0.03 **a**	36.36 ± 3.32 **b**	1.44 ± 0.09 **a**	54.08 ± 3.72 **b**	3.44 ± 0.33
	*p*-value	ns	ns	ns	ns	ns	ns	ns	ns	ns	ns	ns	ns	ns	ns	ns	0.078	0.036	0.058	0.026	ns
2019	Severe Water Stress ± SD	0.92 ± 0.16	0.06 ± 0.01	0.58 ± 0.20	0.04 ± 0.01 **b**	0.18 ± 0.03	0.01 ± 0.00	2.07 ± 0.37	0.14 ± 0.02 **b**	3.98 ± 0.53	0.50 ± 0.12	0.38 ± 0.07	0.06 ± 0.01	3.13 ± 0.37	0.35 ± 0.07	2.15 ± 0.20	0.17 ± 0.04	24.06 ± 2.89	2.24 ± 0.14	33.71 ± 3.67	3.33 ± 0.36
	Moderate Water Stress ± SD	1.07 ± 0.26	0.07 ± 0.01	0.76 ± 0.22	0.05 ± 0.01 **a**	0.20 ± 0.04	0.01 ± 0.00	2.42 ± 0.55	0.16 ± 0.03 **a**	4.52 ± 1.01	0.48 ± 0.11	0.45 ± 0.12	0.06 ± 0.02	3.48 ± 0.71	0.34 ± 0.06	2.43 ± 0.41	0.19 ± 0.04	25.55 ± 4.33	2.29 ± 0.08	36.43 ± 6.37	3.37 ± 0.20
	*p*-value	ns	ns	ns	0.074	ns	ns	ns	0.081	ns	ns	ns	ns	ns	ns	ns	ns	ns	ns	ns	ns
Year		<0.0001	ns	<0.0001	<0.0001	0.003	<0.0001	<0.0001	<0.0001	<0.0001	<0.0001	0.034	<0.0001	<0.0001	0.001071	ns	<0.0001	<0.0001	<0.0001	<0.0001	<0.0001
Year × Zoning		ns	ns	ns	ns	ns	ns	ns	ns	ns	ns	ns	ns	ns	ns	ns	ns	ns	ns	ns	ns

*^a^ANOVA to compare data (p-value indicated); letters within columns indicate significant mean separation according to Tukey’s test.*

For skin anthocyanins, there was no difference observed in total delphinidin, cyanidin, or petunidin on neither per berry basis nor per mg dry skin matter basis in either year (Table 2). There was lower total peonidin content per berry in the severely water stressed zone. The most abundant anthocyanin derivative malvidin had a higher concentration in skin dry matter in the severely water stressed zone in 2018. However, this difference in malvidin was reversed when compared on a content per berry basis, where malvidin concentration was lower in skin dry matter in the severely water stressed zone. There was no significant differences observed in any of the anthocyanin derivatives in the second season.

**FIGURE 7:**
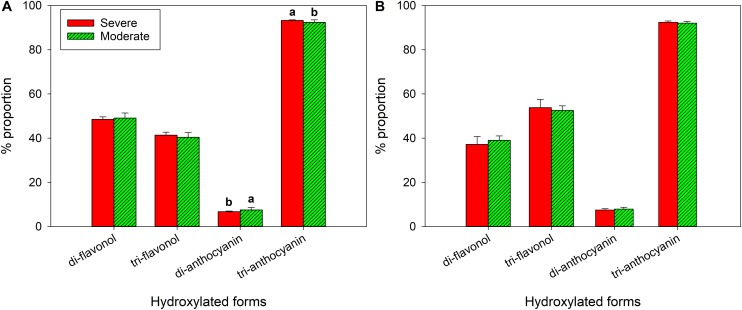
Hydroxylation of flavonols and anthocyanins at harvest of a Cabernet Sauvignon vineyard as separated by plant water status zoning in Oakville, CA in **(A)** 2018 and **(B)** 2019.

In 2018, there was no difference in di-, tri- hydroxylated flavonols (Figure 7A). There was higher proportion of tri-hydroxylated anthocyanins in the severely water stressed zone compared to the other zone, but di-hydroxylated anthocyanin proportion was lower. In 2019, there were no differences between the two water status zones in either flavonol or anthocyanin hydroxylated forms (Figure 7B).

### Relationship Between Proximal Soil Sensing and Physiological Indicators

**FIGURE 8:**
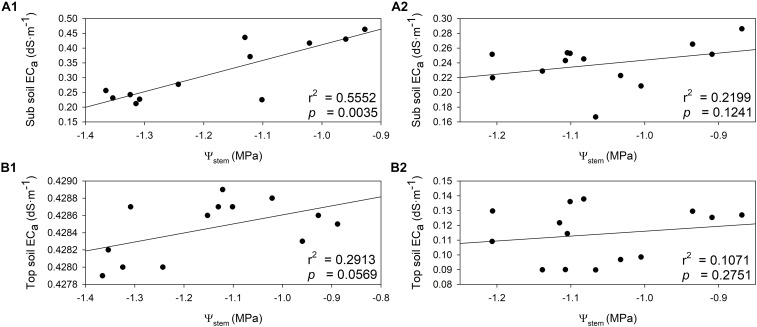
Relationship between stem water potential integrals (Ψ_*stem*_) and soil electrical conductivity (EC_a_) in two soil depths assessed by EM38 in 2018 and 2019. **(A)** 2018, **(B)** 2019, (1) sub soil EC_*a*_ (0.75 – 1.50 m), (2) top soil EC_a_ (0 – 0.75 m).

The relationships between Ψ_*stem*_ and soil EC were investigated in both years. Soil EC values increased when the plant water status was more positive (Figure 8). In 2018, there was a direct and positive relationship between sub soil EC and Ψ_*stem*_ integrals (Figure 8A1, *r*^2^ = 0.5552, *p* = 0.0035). A similar relationship was evident between top soil EC and Ψ_*stem*_ integrals in 2018, albeit not as strong as sub soil EC (Figure 8A2, *r*^2^ = 0.2913, *p* = 0.0569). In 2019, sub soil EC had a moderate linear correlation with Ψ_*stem*_ (Figure 8B1, *r*^2^ = 0.2199, *p* = 0.1241). There was only a weak linear correlation between top soil EC with Ψ_*stem*_ (Figure 8B2, *r*^2^ = 0.1071, *p* = 0.2751). These two correlations were not statistically significant in 2019.

**FIGURE 9:**
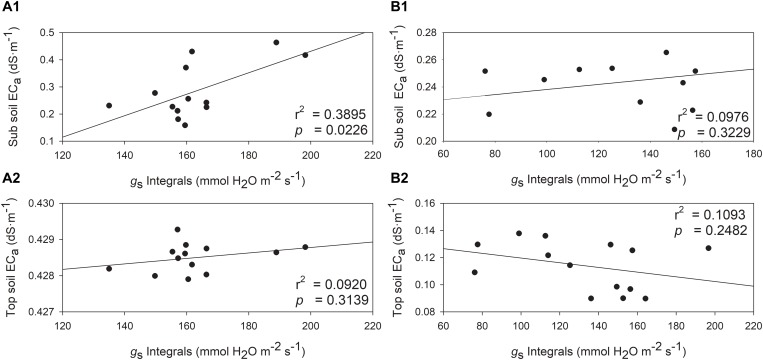
Relationships between stomatal conductance integrals (*g*_*s*_) and soil electrical conductivity (EC_a_) in two depths assessed by EM38 in 2018 and 2019. **(A)** 2018, **(B)** 2019, (1) sub soil EC_a_ (0.75 – 1.50 m), (2) top soil EC_a_ (0 – 0.75 m).

The relationships between *g*_*s*_ integrals and soil EC was also investigated. The soil EC values would increase when higher stomatal conductance was measured, but one exception was observed with top soil EC in 2019 (Figure 9). In 2018, *g*_*s*_ integrals and sub soil EC were directly and positively related (Figure 9A1, *r*^2^ = 0.3895, *p* = 0.0226). Although the top soil EC and *g*_*s*_ integrals showed a similar trend, they were not directly related (Figure 9A2, *r*^2^ = 0.0920, *p* = 0.3139). In 2019, sub soil EC displayed a similar trend with *g*_*s*_ integrals, however, the relationship between them was not significant (Figure 9B1, *r*^2^ = 0.0976, *p* = 0.3229). The top soil EC was not related to *g*_*s*_ integrals in 2019 (Figure 9B2, *r*^2^ = 0.1093, *p* = 0.2482).

## Discussion

### Soil Characteristics, Soil EC and Plant Water Status Relationships in Space

Soil texture can play a critical role in determining soil water holding capacity, total transpirable soil water, and plant water status (Pellegrino et al., 2005; Tramontini et al., 2013). The severely water stressed zone had higher a proportion of silt and clay (Table 3), but a lower proportion of sand and gravel at the depths of 0.75–1.5 m. It had been shown that sandy soil could contribute to more accessibility of soil water content to the plants than clay soil when only soil texture was considered in the scenario (Tramontini et al., 2013). We attributed this factor to one of the possible reasons why one water status zone was directed toward greater water stress in the plants than the other. However, the same study showed that having more gravels in soil would impose more water stressed conditions with more negative plant water potential and lower stomatal conductance. Our results contradicted this condition where the severely water stressed zone had less gravel proportion than the other zone. We attributed this to the finding that the proportion of gravel between the two fHZs not being different enough to allow this factor to affect water availability.

**TABLE 3:** Soil characteristics assessed at field capacity in 2019 of Cabernet Sauvignon as separated by plant water status zoning in Oakville, CA^a^.

		OM	Sand (%)	Silt (%)	Clay (%)	Gravel (%)
Sub soil (0.75 – 1.50 m)	Severe Water Stress ± SD	1.14 ± 0.07	29.00 ± 7.95 **b**	33.67 ± 3.20 **a**	37.33 ± 6.15 **a**	10.32 ± 3.60 **b**
	Moderate Water Stress ± SD	1.14 ± 0.19	41.63 ± 12.25 **a**	28.25 ± 4.65 **b**	30.13 ± 7.86 **b**	14.12 ± 5.79 **a**
	*p* value	ns	0.026	0.031	0.063	0.086
Top soil (0 – 0.75 m)	Severe Water Stress ± SD	1.93 ± 0.27	41.83 ± 2.48	31.17 ± 1.83	27.00 ± 1.67	13.52 ± 2.66
	Moderate Water Stress ± SD	1.93 ± 0.31	40.00 ± 9.56	29.25 ± 4.03	30.75 ± 7.03	14.84 ± 3.67
	*p*-value	ns	ns	ns	ns	ns

*^a^ANOVA to compare data (p-value indicated); Letters within columns indicate significant mean separation according to Tukey’s HSD test.*

Previous studies postulated that installing pressurized irrigation systems may ameliorate the natural spatial variability originating from the soil (Chaves et al., 2010; Rogiers et al., 2011). In our work, irrigation was scheduled and applied uniformly throughout the whole growing season in both years. Still, the plant water status was consistently separated between the two water status zones in both years. This aligned with some conclusions made from our previous work, and further corroborated that with uniform irrigation regimes, the plant water status within one vineyard would not necessarily be uniform (Verdugo-Vásquez et al., 2016; Brillante et al., 2017). Especially with extreme weather conditions being prevalent such as heat waves or more than three times of the normal precipitation amount falling on this vineyard, the spatial variability of the soil would still have a dominant effect on plant development and inevitably reveal the pre-existing various characteristics from the soil.

There were many factors that may alter water availability toward plants. Soil electrical properties can reflect many soil characteristics, including soil texture, soil water content, and soil salinity (Bushman and Mehalick, 1989; Bittelli, 2011; Brillante et al., 2015). This approach had already been applied for soil water content and salinity assessment (Brevik et al., 2006; Brunet et al., 2010; Peralta and Costa, 2013; Brillante et al., 2014). In previous studies, soil electrical properties combined with machine-learning algorithms, was utilized as a useful tool to assess plant available soil water (Brillante et al., 2015, 2016c). Also, it can be used as a baseline to immediately identify the variability in vineyard soils, which can direct soil survey with more focused sampling strategies (Bonfante et al., 2015). In our study, soil EC was assessed on the dates close to harvest to validate the possibility of a simple and direct correlations between soil EC and season long plant water status. Soil EC had a moderate to strong correlation with long-term plant water status Ψ_*stem*_ integrals in both sub and top soil in the first season. The same trend in these relationships were also observed in the second season even though the correlations were not statistically significant. Sub soil EC also showed a significant correlation with *g*_*s*_ integrals in the first year, which had been closely associated with plant water status in previous studies (Costa et al., 2012; Brillante et al., 2017). According to previous research, different varieties responded to water stress differently in terms of controlling stomatal conductance (Rogiers et al., 2011; Pou et al., 2012). Many cultivars did not respond to plant water status as instantly as some others did since stomatal closure would be maintained by accumulated abscisic acid (ABA) under drought (Tombesi et al., 2015). Top soil EC showed a negative relationship with *g*_*s*_ integrals, which did not correspond to the relationship between the two parameters in 2018, nor reflect the plant water status by Ψ_*stem*_ integrals observed in 2019. Previous research had suggested that ground-truthing the soil samples were necessary to interpret the soil EC assessment (Morari et al., 2009). Nevertheless, vineyard delineation based on soil electrical properties were still useful to identify the variability in the soil, and plant physiological and chemical properties derived from it (Bramley et al., 2011a; Tagarakis et al., 2013). These results provided the evidence that proximal sensing soil EC could be a plausible and manageable way to assess spatial variation of plant water status.

Due to the natural spatial variability within vineyards, the noticeable spatial non-uniformity in plant water status was reported previously (Brillante et al., 2016b, 2017). The spatial variability in plant water status stemmed from the highly variable soil characteristics within the vineyard according to previous studies (Grote et al., 2010; Taylor et al., 2010; Brillante et al., 2016b). In our previous work we reported that the spatial variability in plant water status altered the leaf gas exchange (Brillante et al., 2017). A more positive plant water status would increase the leaf stomatal conductance and also increase the net carbon assimilation until the plant reaches its photosynthetic capacity (Flexas et al., 2010; Costa et al., 2012). Although we measured and report these within the confines of study, they were not consistently evident in both years. We attributed this lack of consistency to the highly variable precipitations received at the research vineyard.

### Yield Components

Plant water status had been reported to be one of the main factors affecting grapevine yield components in previous studies, where higher water stress could decrease berry weight, berry skin weight, and yield per vine (Castellarin et al., 2007a; Santesteban et al., 2011; Bonada et al., 2013). In our study, we measured less berry weight and berry skin weight in the severely water stressed zone in 2018, but not in 2019. However, the plant water status between the two fHZs were consistently separated in both years. Thus, the inconsistency might be due to the smaller difference in Ψ_*stem*_ integrals between the two fHZs in 2019, where 0.18 MPa was observed from the start of the season to harvest and 0.16 MPa from veraison to harvest (data not shown) compared to 0.27 MPa in 2018. This was also attributed to the great variation in precipitation received in both years as indicated by the significant Year effect presented herein. We observed no difference in yield per vine between two water status zones, which was also observed previously (Acevedo-Opazo et al., 2010; Brillante et al., 2017). A lacking of water stress severity, and the plant water statuses between the two fHZs were not significantly dissimilar could be the reason that no detrimental yield loss was observed in neither years.

In small plot trials water stress was effective in altering plant canopy development, and leaf area was directly related to canopy microclimate (Intrigliolo and Castel, 2010; Keller et al., 2016; Kraus et al., 2018). Previous studies had also shown that canopy microclimate had a determining role in altering berry chemistry biosynthesis (Cook et al., 2015; Yu et al., 2016). In our current work, we did not observe differences in leaf area even though the plant water status was consistently separated throughout the two seasons. Leaf area to fruit ratio was used to characterize the source-sink relationship (Kliewer and Dokoozlian, 2005; Zufferey et al., 2012). Previous research suggested that to reach the maximum level of maturity, a leaf area to fruit ratio between 0.8 to 1.2 m^2^/kg was required for a single-canopy trellis system (Kliewer and Dokoozlian, 2005). In our study, there was no difference of leaf area to fruit ration between two water status zones and the first year had overall leaf to fruit ratio lower than 0.8 m^2^/kg consistent with previous findings that mechanically managed vineyards in warm regions may ripen fruit to technological maturity at lower values (Kurtural et al., 2019).

### Berry Primary Metabolisms

The severity of water stress in grapevine is a determining factor in directing berry primary metabolism according to previous studies (Basile et al., 2011; Santesteban et al., 2011). Moderate water stress could lead to a more advanced maturity, which would result in higher TSS and lower TA (Brillante et al., 2017). However, severe water stress may cause a delay in berry development (Korkutal et al., 2011; Martínez-Lüscher et al., 2015). There was no difference observed in any of the berry primary metabolites in either year, except TA in 2019. The difference in plant water status was not greatly different enough to solicit a difference in the berry maturity levels between the two fHZs. Likewise, previous research indicated that *A*_*n*_ was directly related to final TSS accumulation, where higher *A*_*n*_ led to higher TSS (Brillante et al., 2016a). However, our study did not give consistently evident separations in net carbon assimilation. In previous studies, TA reduction after veraison was usually used as an indicator of berry maturity aside from TSS accumulation (Dai et al., 2011). Even though both seasons did not have water stress great enough to alter the TSS accumulation, there was a greater advancement in berry maturity in 2019 compared to 2018. We attributed this to the greater GDD accumulation during 2019 as well as the greater amount of precipitation received. Furthermore, in the first year, leaf area to fruit ratio was lower than the lower limit of the 0.8 m^2^/kg requirement. This ‘over-cropping’ condition might have contributed to the lower TSS in the first year, causing the fruits had a less advanced development to reach the maximum level of maturity. Therefore, the difference observed in TA could be because the malate metabolism became more sensitive toward water stress at a more advanced ripening stage in 2019 (Cramer et al., 2007; Sweetman et al., 2014).

### Berry Secondary Metabolisms – Skin Flavonoids

Water stress had been reported to have direct effects on both flavonoid biosynthesis, and flavonoid concentration due to berry dehydration (Castellarin et al., 2007a; Bondada and Shutthanandan, 2012; Brillante et al., 2017). Previous studies had shown that moderate water stress can enhance berry skin flavonoid accumulation as well as the concentration (Bucchetti et al., 2011; Martínez-Lüscher et al., 2014a). However, when the severity of water stress increased even further, the degradation of flavonoid compounds could be more pronounced (Brillante et al., 2017). According to previous research, anthocyanins were more sensitive toward water stress than flavonols (Castellarin et al., 2007a). It was also shown that flavonol accumulation could also be altered by different water deficit irrigation regimes (Zarrouk et al., 2012). However, some studies had pointed out that flavonols were more dominated by solar radiation than water stress, and they were particularly sensitive toward UV-B (Martínez-Lüscher et al., 2014a, b). In our study, higher quercetin and total flavonol content per berry were observed in the moderately water stressed zone. Although the leaf area comparisons within these two fHZs did not reveal a significant difference, we attribute the difference in flavonol profiles to spatial variability of soil water supply as corroborated in our recent work (Martínez-Lüscher et al., 2014b).

The increasing content of total skin anthocyanins with water stress in previous studies agreed with our results in 2018, where the fruits from the severely water stressed zone showed a higher total anthocyanin concentration in the skin tissues (Brillante et al., 2017). This effect was not observed on the content per berry basis. When compared between the two water status zones, moderate water stress led to higher peonidin, malvidin content per berry, yet severe water stress lead to higher malvidin and total skin anthocyanin concentration in skin dry matter. This discrepancy in the weight basis and per berry basis was observed in previous studies (Ginestar et al., 1998; Kennedy et al., 2002). It could be due to the enhanced anthocyanin concentration relative to the skin tissue masses, but less total amount of flavonoid compounds accumulated in each berry in 2018. We could not rule out that the severe water stress may had led toward a greater anthocyanins degradation, or the overall berry development was slightly slowed down like our yield component results presented, causing less flavonoids accumulated. Additionally, the second year showed an overall lower anthocyanin per mg dry skin basis compared to the first year. Previous research showed that an advanced maturity would initiate anthocyanin degradation (Brillante et al., 2017). After around 23°Brix, anthocyanin degradation (on both per g of berry mass basis and per berry basis) would be exacerbated (Martínez-Lüscher et al., 2017, 2019). However, the degradation might not be the only reason to thoroughly explain the phenomenon between these two years since anthocyanin content per berry was higher in 2019 compared to 2018. We observed greater berry skin weight but lower berry weight in 2019 than 2018. Thus, one possibility was that the effect of the advanced berry development on berry physical characteristics overrode the effect on berry skin chemical characteristics. The total anthocyanin content of the whole plant might be lower due to the degradation in 2019 than 2018, but the berry numbers were also lower as observed. The average anthocyanin content per berry can still be higher in 2019 than 2018.

As corroborated by previous studies, the severely water stressed zone had higher proportion of tri-hydroxylated and lower di-hydroxylated anthocyanins when compared to moderately water stressed zone (Castellarin et al., 2007b; Martínez-Lüscher et al., 2014a; Brillante et al., 2017). Previous work provided evidence that, F3′5′H can be upregulated in the flavonoid biosynthetic pathway with moderate water stress (Castellarin et al., 2007a), which would result in increasing hydroxylation level on the B-ring of flavonoid skeleton. Additionally, tri-hydroxylated anthocyanins were more stable against oxidation or degradation than di-hydroxylated forms (Mori et al., 2007), which could be another reason besides F3′5′H upregulation to have a higher proportion with severe water stress. Although the possibly affected transcription factors F3′H and F3′5′H were shared to produce both flavonols and anthocyanins in the same pathway branches, tri- or di-hydroxylated flavonols along were not disparate between the two fHZs in our study.

## Conclusion

Recent precision viticulture studies had proposed that vineyard delineation can be a plausible approach to monitor and manage spatial variability present in the vineyard (Peralta and Costa, 2013; Tagarakis et al., 2013; González-Fernández et al., 2017). Being a critical physiological parameter, plant water status was able to successfully capture the spatial variability in the final berry chemistry in previous research (Brillante et al., 2017, 2018), and it was further studied in this specific study. Study presented here in provided evidence that the spatial variability within the vineyard can be apparent in plant physiology and berry chemistry. Moreover, our results provided evidence that proximal sensing of soil EC may be a useful tool to connect soil to plant water status, even further to berry primary and secondary chemistry as observed in recent research (Bonfante et al., 2015; Tardaguila et al., 2018; Priori et al., 2019). This fundamental knowledge can contribute to a greater linkage between available sensing technologies and quality-related chemical analysis in precision viticulture research (Matese et al., 2015). The promptness and efficiency of proximal sensing can be transformed into realistic utilization, which can be significantly beneficial in large-acreage vineyards.

## Data Availability Statement

The raw data supporting the conclusions of this article are available on request to the corresponding author.

## Author Contributions

SK designed the project, acquired the funding. RY collected and analyzed the data, wrote the first version of the manuscript. RY and SK interpreted the data, read and approved the final version of the manuscript.

## Conflict of Interest

The authors declare that the research was conducted in the absence of any commercial or financial relationships that could be construed as a potential conflict of interest.
